# Tobacco Cessation Intervention for Young People

**DOI:** 10.7759/cureus.30308

**Published:** 2022-10-14

**Authors:** Avni Gakkhar, Ashok Mehendale, Shivansh Mehendale

**Affiliations:** 1 Community Medicine, Jawaharlal Nehru Medical College, Datta Meghe Institute of Medical Sciences, Wardha, IND; 2 Community Medicine, Jawaharlal Nehru Medical College, Datta Meghe Insititute of Medical Sciences, Wardha, IND

**Keywords:** rct (randomised controlled trials), councelling, behaviour, stop, health, toxicity, quit, smoking, tobacco cessation, tobacco

## Abstract

Most adolescent tobacco control programs focus on preventing consumption, but teen smoking persists. It is uncertain whether adult-specific therapies can assist adolescents in quitting smoking. The rising incidence of smoking in low and middle-socioeconomic countries and the challenges of conducting tobacco cessation programs in these settings (due to increasing population, poor lifestyle, lack of awareness and education, professional stress, and non-compliance) need an emphasis on the scope of trials to conduct tobacco cessation in these settings. In the 11th Five Year Plan, the Indian government introduced a new National Tobacco Control Program, which currently spans 108 districts in 31 states across the country. The objective is to review the randomized control trials of selected individuals held in India and assess and evaluate the effectiveness of the steps taken by the government to help people quit tobacco consumption. These programs are important because of the hazards and impact they have on the public health indices of the nation. The government has taken steps like prohibiting smoking in public areas and banning sources. Several programs, particularly those that employed group counselling, included a range of approaches that proved beneficial in helping young people quit smoking. The RCTs mentioned are psychosocially followed by behavioral and pharmacological therapies. The challenges faced are not having participatory health care, motivating tobacco users to quit even in the short-term, adequate coverage; barriers at a different level of implementation (at a regional, state, or national level); and interference by the tobacco industry should be eliminated. The currently functioning programs in India are the National Tobacco Control Program, the National and three Regional Quitlines and mCessation.

## Introduction and background

**Figure 1:**
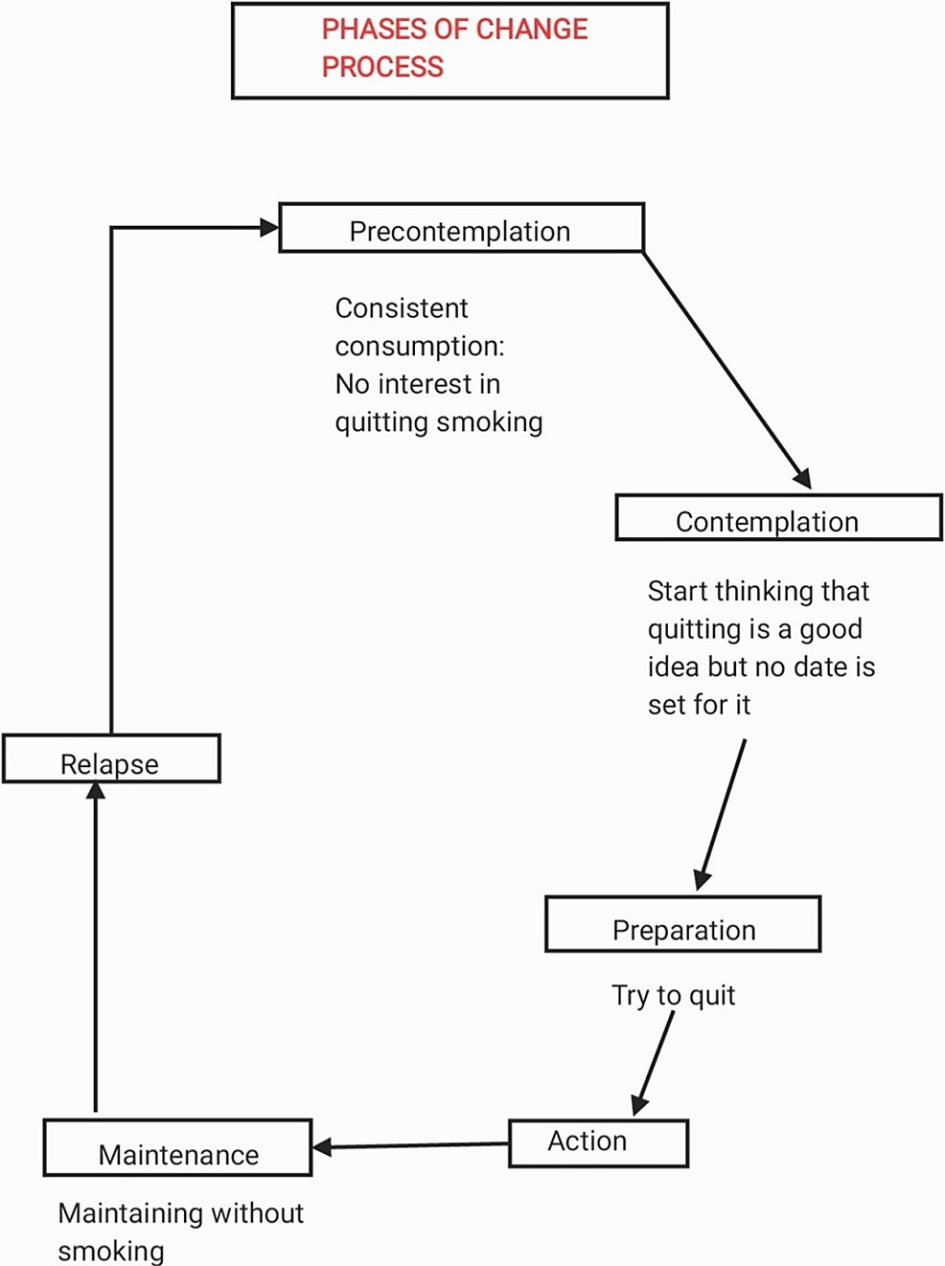
Phases of the change process.

**Figure 2:**
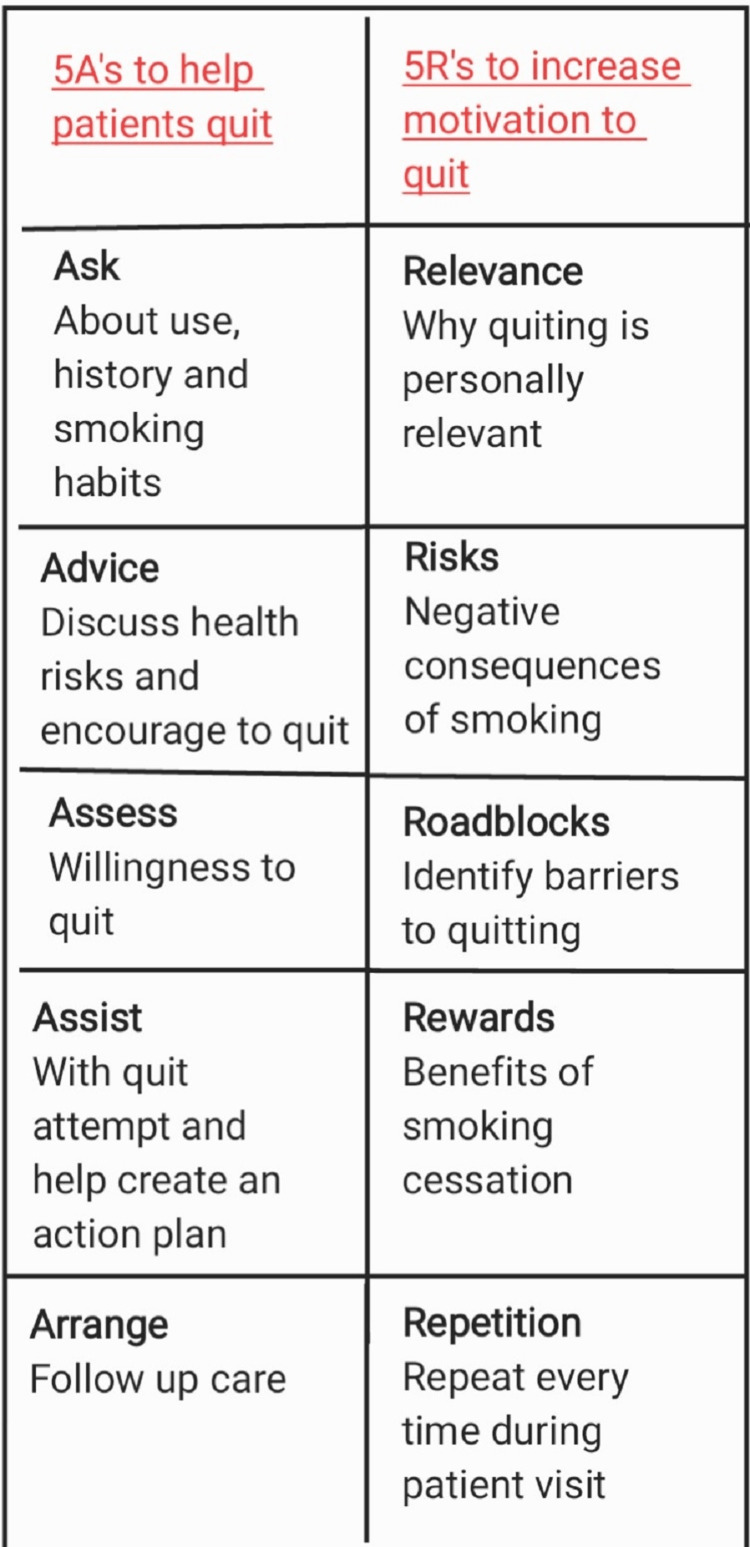
Smoking cessation - 5 A's and 5 R's

Smoking habits in young adults are associated with years of smoking. Most young adults start as occasional smokers, but their dependency grows and they become chronic smokers. This is why the tobacco industry focuses not only on making individuals start smoking but also on motivating occasionals to smoke regularly. The factors that lead to the initiation of smoking are primarily professional or career stress, family issues, unstable mental health, which is gaining all the attention these days, and also financial state since India is still a developing country. Stronger designs were used for psychosocials. Randomized controlled trials (RCTs) were conducted in China, India, Brazil, Malaysia, and Thailand [1]. Apart from tobacco paper and a filter, the production of a cigarette involves a wide range of products. Flavourings, enhancers, humectants, sweeteners, and compounds of ammonia are tobacco additives [2]. Several researchers have questioned tobacco companies who claim that substances used to glamourize tobacco do not increase toxicity or make it more desirable. Tobacco additives cause an increase in formaldehyde levels and small changes in other smoke contents [3]. According to various research, the impact of tobacco additives on the harmfulness of cigarette smoke is yet unknown [4]. The rate of deaths due to smoking (most fatal being lung cancer due to smoking and oral cancer due to tobacco chewing) can be reduced through aggressive policy measures [5]. We can also learn about the effectiveness of the programmes by conducting research among adults who have stopped smoking because of the interventions. People who continue to smoke can help us improve or adjust the interventions from less to more effective [6]. It is unknown how smoking rates change, what factors relate to present smoking, and whether changing with time is linked to more regular cigarette use among young adults [7]. Respondents who said they smoked at least 100 cigarettes in their lifetime and smoked every day or on certain days were considered current smokers. Former smokers included ex-smokers and people who had smoked 100 or fewer cigarettes [8]. Smoking is a scientifically proven risk factor for several problems, including increased blood pressure and sugar levels, cardiac disease, chronic obstructive pulmonary disease (COPD), and cancer. It is the second leading cause of early death and disability [9]. Tobacco control interventions, such as tobacco taxation, smoking bans in public places, and smoke-free zones, have significantly enhanced during the last decade. However, global cigarette smoking prevalence stayed high in 2015, with 25% of men and 5.4% of women smoking [10]. Nicotine was identified in the herbaceous plant Nicotiana tabacum, which is native to tropical and subtropical America but is now commercially cultivated worldwide. In developing countries, young adults are most likely to start smoking, usually around adolescence. Although chronic smokers bear most of the illness burden, there are various reasons why smoking cessation therapies that are effective in new/younger smokers are especially useful (because these young smokers eventually become chronic smokers in the future) [11]. Many of the negative health impacts of smoking can be avoided by quitting when you are young; there is no change in life expectancy if you quit early. There is evidence that people who begin smoking at a young age and continue to smoke into maturity are more prone to disease than those who start later in life, facing lung damage and bowel damage. Cancer and cervical precancerous lesions are all hazards [12]. The use of different tobacco products is concerning since it is linked to increased nicotine dependence symptoms, an increased risk of body problems (e.g., infarction), and a lower chance of quitting [13]. There's also evidence that many teen smokers like to quit after only a few weeks of smoking. Smoking cessation may be challenging for young people with mental health or behavioral disorders such as conduct disorder, emotional disease, or attention deficit hyperactivity disorder [14]. Because of the devastating physical, social, and economic consequences of smoking, it is critical to observe and study the influential components of smoking and the goals for smoking cessation (Figure 1). There is a sequence of steps that help in smoking cessation that are mentioned in Figure 2.

Harmful effects of passive smoking

Passive smoking is as harmful as active smoking. Environmental tobacco smoke (ETS) is the term used to characterize tobacco combustion products inhaled by nonsmokers in the proximity of burning tobacco. Among adults with preexisting health conditions such as allergies, chronic lung conditions, and angina, the symptoms of these conditions are exacerbated by exposure to ETS [15]. Sidestream smoke contains higher concentrations of ammonia, benzene, nicotine, carbon monoxide, and many carcinogens [16,17]. An increased incidence of lower respiratory tract infections like bronchitis, asthma, and pneumonia in children of smokers has been reported. Children of parents who smoke are not only sick more frequently from respiratory ailments but are also more likely to have impaired lung growth as they develop, which may increase the risk of chronic airflow obstruction as an adult [17,18]. Researchers reported that ETS increased heart rate, blood pressure, and oxygen consumption [19]. Passive smoking could increase the risk of some diseases among children, especially bacterial infections (e.g., lower respiratory infections in infancy, middle ear disease in children, invasive meningococcal disease in children, allergic diseases in children, and childhood asthma) [20]. Preliminary data from a prospective study conducted in San Diego indicated that the nonsmoking wives of smoking husbands have an increased risk of dying from ischemic heart disease [21]. Major prevention can be done by prohibiting smoking in public areas like airports, restaurants, movie halls, cafes, etc., and reducing the source of direct smoking.

## Review

Tobacco addiction treatment for young people has mostly focused on primary prevention. The review of programs initiated by schools and reviews of mass media assessment and treatment, as well as community interventions, are among them for decreasing the reach by preventing the illegal sale of tobacco [22]. Because knowing the smoking pattern in young people is important for smoking prevention, several previous types of research have concentrated on this cohort. However, those studies primarily aimed at identifying factors associated with young adults' smoking behaviors or comparing characteristics between young people and people of other generations; as a result, less is known about dissimilarities among adults [23]. More precisely, it is unknown how smoking behavior keeps evolving, what factors contribute to current smoking, or even if growing older is linked to greater cigarette usage among young adults.

RCTs conducted in India

Survey Done in Delhi

The study was conducted on participants of the desired age group chosen from localities with high smoking rates. The study was conducted after assessing common socioeconomic and tobacco consumption factors at the beginning. Their age, sex, and marital status were added, and participants were asked if they were employed or not; their education levels (elemental education vs. not); social class (lower caste vs. other); and family earnings (five thousand vs. five thousand rupees/month). A survey of young individuals in Delhi's industrialized low-earning regions was conducted to establish a suitable sampling frame for this study (Table 1). Longitudinal studies that follow patients and parents through follow-up clinics are needed so that supplies and products continue until cessation is completed, as well as to obtain health metrics of the children [24]. Since chawls/blocks in the study area are divided into two types of settlements (government colonies and unofficial kitchen homes known as Jhuggi-Jhopri) were included. Before randomizing people for interventional or control groups, the eligible tobacco users from each group were selected. A computer-based random selection of adults was made so that individuals from each community could take part. Sixteen groups (eight from every community type) were assigned randomly to the intervention and control groups based on the random sequence. While people who participated were blind to their allotment because it was a group-randomized study, hiding the assigned task from the research team was impractical [25]. The interventions used were a single 15-minute session of smoking - cessation therapy as well as brief instructions about practicing two yogic breathing methods. The quit counseling included coping techniques, medical advice, social support, and relapse prevention, all of which have been shown to improve stop rates. 'Kapaalbhati' (deep inhalation and exhalation) and 'Anulomvilom' (alternate nostril breathing), two easy-to-learn and practice breathing exercises, were advised because they are widely accepted and have scientifically helped people overcome withdrawal [26]. The research team used a written standard operating procedure, including a message for quitting advice and a standard video on how to do yoga and breathing practices [27]. The group that received behavioral support and cigarette cessation drugs (nicotine patches) had mixed outcomes. Still, group-based therapies, which included child counseling with their parents, relatives, and campaigns, had a positive impact [11]. Although a control group with no tobacco use therapy would have been ideal for assessing the full intervention impact, this was considered immoral. As a result, only one control meeting was used, with extremely short quitting advice. This lasted an average of one minute and included voice notes and audio about tobacco use's dangers and tips on quitting.

**Table 1:** Details about the RCTs conducted in India. RCT: randomized controlled trial [1].

Location	No. of participants	Inclusion criteria	Exclusion criteria	Study duration
Gurgaon	124	Expressed self-intent to quit	NA	12 weeks
Chandigarh	156	Sputum smear-positive pulmonary TB patients; males and females; aged 15 years and above	Smokeless tobacco users	6 months
New Delhi	237	Use of smokeless tobacco each day for the past year (confirmed with urinary cotinine assessment; 50 ng/ml); age over 18; residing within 60 miles of New Delhi	Current cigarette use (confirmed with breath carbon monoxide [CO] >10 ppm); Current or planned use of tobacco cessation treatment; current use of cocaine, marijuana, or opioids or current consumption of 25 alcoholic drinks/week	12 weeks
Kerala	928	Males; age group of 18–60 years; had reported using at least one cigarette/bidi daily during the study period	Females; subjects who could not speak; mentally disabled; terminally ill patients.	12 months

Survey done in Bihar

In 2009, 72 schools were selected to participate in an RCT where school teachers who smoked were divided into intervention and control groups. Eight teachers were selected from each school [28]. The intervention aimed to promote change in five mediating mechanisms: risk perceptions, motivation to change, social norms and role models supporting tobacco control, self-efficacy and skills for quitting, and support to quit. The study duration was seven months. Thirty days quit rate was 50% in the intervention and 15% in the control group. A survey after nine months showed a six-month abstinence rate of 20% and 5%, respectively [29].

Interventions

Clusters of young adults are given psychological therapy where they are taught about ways to stop smoking and a school-wide ban on cigarette advertising [30]. This study aimed to see if a volunteer-based inpatient tobacco cessation program could be effective in consumption, maintenance, and quitting. The key finding of this study was that using volunteers to reach inpatient tobacco users is a practical technique, as evidenced by the 72% of tobacco users who visited and the 97% who accepted the visit [31]. Receiving helpline services increased the chances of quitting after discharge, while having a volunteer visit did not [32]. These findings support the practicability of using hospital-oriented volunteers and the value of giving volunteers nicotine replacement therapy and interacting with helpline programs to aid in post-discharge cessation. These findings support the practicability of using health center-based volunteers and the importance of giving inpatient replacement therapy and interacting with helpline programs to help with cessation. While many researchers have utilized health professionals to provide inpatient cessation programs, this is the first to use trained bedside trainers [31-33]. Thirty-eight percent of patients were sent to the helpline with a short volunteer visit. Thirty-six percent accepted assistance, similar to other helpline referral studies that used a comforting helpline referral or quick telephone intervention. Patients who received replacement therapy while in the hospital were twice as likely to accept a helpline referral, and patients who received helpline services had a threefold increase in the likelihood of not using tobacco three months after their release [31-34]. Prior studies have shown the success of helpline numbers in assisting patients in making a stopping attempt, more rigorous inpatient tobacco-free programs, and community-based initiatives that use trainer cessation services (Table 2) [35]. Pharmaceutical therapy was paired with counseling in both psychosocial/pharmacological RCTs. One RCT, for example, used varenicline, who were getting behavioral counseling. Varenicline (43%) had significantly higher self-reported abstinence than placebo. In both younger and older young adults, the non-college-educated group's current smoking rate was more than that of the college-passed population. A large percentage were comparatively poor, as defined by family earnings, and around 50% were from the lower economic strata. Most of them had been using tobacco products for at least 20 years and were mostly addicted. A few had tried to stop in the previous year, with prior attempts lasting up to two months, but only a few had used any quit help. The majority of the respondents felt confident in their capacity to quit. Even tiny changes in cigarette usage can have clinical significance, despite the small effect observed. However, if such a strategy were to be applied all over India, it would almost certainly result in the abolition of millions of cigarette smokers each year. We demonstrated the positive impact of getting nicotine replacement therapy during hospitalization [34-36]. Patients with good experiences with nicotine replacement therapy may be more likely to continue using it and be clean and sober; the helpline offers free nicotine replacement therapy and support to help them achieve precisely that [37]. Table 2 shows interventions used in tobacco cessation

**Table 2:** Results of interventions in Indian setting RCT: randomized controlled trial [1]

Interventional RCTs	Techniques used	Results
Psychosocial RCTs (70%)	30 minutes individual counselling, group counselling, health education	Had greater impact
Behavioural RCTs (19%)	Yogic exercises (Annulom vilom, Kapaalbhati)	Positive impact
Pharmacological RCTs (8%)	Bupropion, Varencline, Nicotine patch therapy	Non-compliance can be an issue

## Conclusions

The results are most promising for group-based behavioral therapies, although evidence for all intervention modalities is still limited. For this group of smokers, well-designed, sufficiently powered, randomized controlled trials of treatments are still needed. The majority (70%) of trials' results were psychosocial, with behavior change (19%) and psychosocial/pharmacological (19%) following closely behind (8%). Most psychosocial studies included therapy and strategies such as 30-minute individual and group counseling. Physician-led health education, pharmacological therapy using nicotine patches or varenicline, health education in areas with high smoking rates, and patient talks with community health workers were all examples of psychosocial RCTs. Various culturally unique variations of psychosocial/behavioral RCTs included counseling and yoga. In one of these trials, people who did yoga had a higher chance of abstinence than those who simply received behavioral counseling. Manual workers had a smoking prevalence of over five times that of non-manual workers. Younger individuals exposed to smoking habits in the family were more likely to smoke. Finally, older or young individuals who were under a lot of stress were 1.5 times more prone to smoking than those who were not. To conclude, we have demonstrated a strong effect of a potentially affordable, large-scale, self-implicated, culturally sensitive tobacco cessation intervention in lower-middle-income countries (LMIC). In a study of tobacco users from less-earning communities in India, a low-cost interventional affordable single quitting advice session with yoga breathing exercises increased abstinence rates for six months at least. It maintained confirmed stoppage rates fivefold compared to very brief quitting counseling alone. The 2% improvement in complete stoppage rates is comparable to that seen in high-income countries (HIC) with other low-intensity interventions. These results also look good compared to older analyses of more rigorous interventions in LMICs, which roughly doubled short-term tobacco stoppage rates.
